# pH-responsive polymeric vesicles from branched copolymers†

**DOI:** 10.1039/c9ra08703f

**Published:** 2019-12-11

**Authors:** Jinglun Zhou, Linlin Li, Weishan Wang, Yang Zhao, Shengyu Feng

**Affiliations:** Key Laboratory of Special Functional Aggregated Materials, Ministry of Education, School of Chemistry and Chemical Engineering, Shandong University Jinan 250100 China fsy@sdu.edu.cn; Shandong Institute for Product Quality Inspection Jinan 250100 China lilinlin1003@163.com; Eco-Benign Plastics Technology Company Limited Jinan 250101 China

## Abstract

A new type of branched copolymer, poly(l-lactide)_2_-*b*-poly(l-glutamic acid) (PLLA_2_–PLGA), based on polypeptide PLGA is synthesized by the ring-opening polymerization (ROP) of *N*-carboxyanhydride of γ-benzyl-l-glutamate (BLG–NCA) with amino-terminated PLLA_2_–NH_2_ and subsequent deprotection. The branched copolymer is characterized by ^1^H NMR, FTIR and GPC measurements. The self-assembly of the copolymers in aqueous media has been systematically discussed. A pyrene probe has been used to demonstrate the aggregated formation of PLLA_2_–PLGA in solution by measuring the critical micelle concentration (cmc). The morphology and size of the micelles have further been studied by transmission electron microscopy (TEM), dynamic light scattering (DLS) and field emission scanning electron microscopy (ESEM). We demonstrated that the Rh of the vesicle is depending on solution pH and salt concentration. The vesicles show good stability with remained shapes and sizes during the lyophilizing process. These vesicles have great potential in the application of drug delivery.

## Abstract 2

A new type of branched copolymer, poly(l-lactide)_2_-*b*-poly(l-glutamic acid), based on polypeptide PLGA is synthesized by the ring-opening polymerization of *N*-carboxyanhydride of γ-benzyl-l-glutamate with amino-terminated PLLA_2_–NH_2_ and subsequent deprotection.

## Graphical abstract

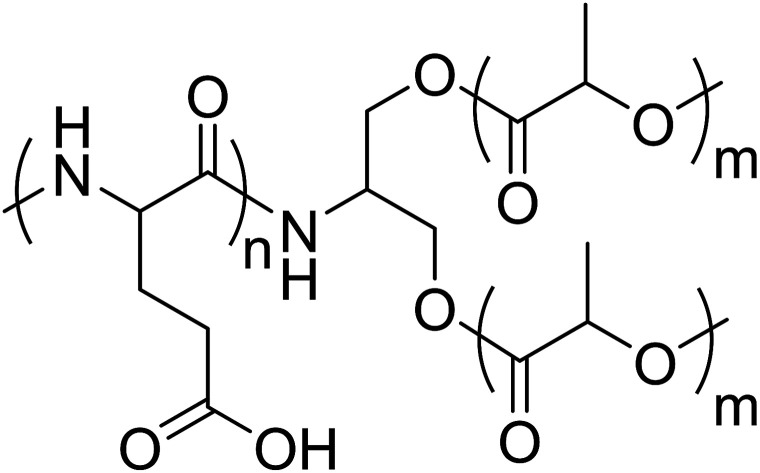

## Introduction

In biology, various kinds of biomacromolecules or biomolecules, including proteins and phospholipids, can spontaneously assemble into vesicles or vesicle-like structures through well-controlled inter- and/or intra-molecular interactions, such as electrostatics, hydrogen-bonding and hydrophobicity.^1,2^ Recent progress in polymer chemistry has facilitated the design and the preparation of amphiphilic membranes (vesicle-like structure) made *via* self-assembly of various novel block copolymers.^3–8^ Self-assembly of block copolymers is a very useful means of creating nanostructured materials with tunable properties, and has attracted considerable attention in material science and biomimetic research.^9–11^ In comparison with the analogous biological membranes, block copolymers membranes possess of enhanced stability and improved mechanical properties because of their longer chains.^12,13^ Many examples of block copolymeric vesicles with different functionalities have been reported, which show potential applications extending from traditional delivery systems to electronics.^14–17^

In the biomedical materials field, copolymeric micelles possess biocompatibility, biodegradability, target specificity, and stability in the body. Poly(l-lactide) (PLLA) is one of the most commonly used relatively hydrophobic and biodegradable polyesters, which has been applied in surgical repair, carriers in drug delivery, and temporary matrixes or scaffolds in tissue engineering due to its biodegradability, biocompatibility, high mechanical properties, excellent shaping and molding properties.^18,19^ A number of polymeric micelles formed from PLA-based amphiphilic diblock copolymers have been investigated in terms of various biomedical applications.^20–26^

Proteins are necessary for human beings. They are known to form α-helices or β-sheets as their fundamental secondary motifs *via* intra- and intermolecular interactions between the functional groups of residual amino acids. Compared to natural proteins, synthetic polypeptides offer more advantages in stability and processability. Great efforts have been made to incorporate proteins or polypeptides into synthetic materials.^27–29^ Compared with those block copolymers without polypeptide blocks, polypeptide-based ones have been shown to provide significant advantages in controlling both the function and supramolecular structure of bioinspired self-assemblies. They can simulate not only the shape of natural vesicles or micelles, but also improve their biological performances. Although polypeptide-containing block copolymers have been available since the 1970s, the work on their self-assembling behavior in aqueous solution are still less reported.

Combining PLLA with peptide blocks can modify the degradation pattern of the polymers because peptidase is required to hydrolyze the peptide bonds. The copolymers consisting of both polypeptides and biodegradable polyesters have been studied rarely.^30,31^ Moreover, they are almost linear shaped copolymer. In this study, we synthesized a novel branched copolymer, poly(l-lactide)_2_-*b*-poly(γ-benzyl-l-glutamic acid) (PLLA_2_-PBLG) by the ring-opening polymerization (ROP) of *N*-carboxyanhydride of γ-benzyl-l-glutamate (BLG–NCA) with amino-terminated polylactide and subsequent deprotection. The branched copolymer may exhibit different micellization behavior as compared to the traditional linear copolymers.^32,33^ The linear copolymers consisting of both polypeptides and biodegradable polyesters usually self-assemble into spheres in aqueous media.^34^ However, due to the branched structure, the obtained copolymers can self-assemble into vesicle structure in aqueous media. Both the charge state and solubility of PLGA blocks are dependent on pH value in solution. Therefore, the self-assembles show pH-dependent behavior as well as salt-dependence.

## Experimental

### Materials

33 wt% solution of HBr in HAc was supplied by Acros. Benzyloxycarbonyl chloride and trifluoroacetic acid were purchased from GL Biochem (Shanghai) Ltd. l-Lactide was purchased from PURAC Biochem by Gorinchem and recrystallized from ethyl acetate for three times. 2-Amino-1,3-propanediol was purchased from Tokyo Chemical Industry Co., Ltd. The hexane and tetrahydrofuran were dried by CaH_2_ to remove water, and purged with dry nitrogen, and then passed through an activated alumina column. All other chemicals were purchased from various commercial suppliers without further purification unless otherwise stated.

### Synthesis of copolymers PLLA_2_–PLGA

The copolymers PLLA_2_–PBLG were synthesized through a modified reported method.^35^ The benzyloxycarbonyl amino group bearing PLLA was first prepared by the ROP of l-lactide in the presence of initiator (2-benzyloxycarbonylamino-1,3-propanediol) and stannous octoate (Sn(Oct)_2_). The benzyloxycarbonyl group on PLLA_2_–NH–Z was further removed by HBr in CF_3_COOH to give PLLA_2_–NH_2_.

The PLLA_2_–PBLG were then synthesized *via* the ring-opening polymerization (ROP) of BLG–NCA initiated by PLLA_2_–NH_2_. The benzyl groups on PLLA_2_–PBLG were removed by reacting with 4 equivalent of HBr (in HAc, *C* = 33%) with respect to γ-benzyl-l-glutamic acid repeat units in CF_3_COOH (0.04 g ml^−1^) at 0 °C for 2 h. The product PLLA_2_–PLGA was precipitated with an excess of diethyl ether to get a white solid and was dried in vacuum at room temperature for 48 h. The sample was purified by dissolution/precipitation in THF/petroleum ether.

### Measurements of the block copolymers

^1^H NMR spectra were measured in DMSO-*d*_6_ at room temperature (25 °C) by an AV-300 NMR spectrometer from Bruker. FT-IR spectra were recorded on a Bio-Rad Win-IR instrument. Gel permeation chromatography/laser light scattering (GPC/LLS) was performed at 50 °C with Tskgel H_HR_ columns (TOSOH BIOSCIENCE) and MZ-Gel SD plus column (50 × 8 mm) using an SSI pump connected to Wyatt Optilab DSP. 0.02 M LiBr in DMF was used as the eluent at a flow rate of 1.0 ml min^−1^. The molecular weights were calibrated against polystyrene (PS) standards. The concentrations of all samples were about 5 mg ml^−1^.

### Preparation of polymer solutions

The block copolymer was first dissolved in *N*,*N*-dimethylformamide (DMF), which was a common solvent for two blocks, with the initial concentration of 2.0 wt%. Then given mount of deionized water was added to the copolymer solution under gentle stirring. To reach an equilibrium, the mixture was stirred overnight. After that, the mixture was further diluted with a large amount of water and was dialyzed against deionized water to remove DMF from the solution.

### Characterization of vesicle morphology and size

#### Fluorescence measurement

A pyrene probe was used to prove the formation of micelles. Steady state fluorescence spectra were obtained by PerkinElmer LS50B luminescence spectrometer. The copolymer solution and distilled water were added consecutively into the volumetric flasks containing pyrene, and the copolymer concentration was from 10^−4^ to 0.4 g l^−1^. The pyrene concentration in each final solution was 6 × 10^−7^ mol l^−1^ (the saturation solubility of pyrene in water at 22 °C). The emission wavelength was 391 nm for fluorescence excitation spectra. The spectra were recorded at a scan rate of 240 nm min^−1^.

#### ^1^H NMR

^1^H NMR spectra were measured in D_2_O at room temperature (25 °C) by an AV-300 NMR spectrometer.

#### Dynamic light scattering (DLS) measurements

DLS measurements were carried out with a DAMN EOS instrument equipped with a He–Ne laser at the scattering angle of 90°. The micelle solution of about 0.4 mg ml^−1^ was passed through a 0.45 μm filter before measurement.

#### Environmental scanning electron microscopy (ESEM) measurements

The ESEM images were recorded with model XL 30 ESEM FEG from Micro FEI Philips. The dilute micelle solution was deposited on a silicon wafer to a very thin layer and dried at room temperature. A thin layer of Au was coated on the sample surface before measurement.

#### Transmission electron microscopy (TEM) measurements

TEM measurements were performed on a JEOL JEM-1011 electron microscope operating at an acceleration voltage of 100 kV. A drop of the dilute aqueous solution was deposited onto a copper grid for about 5 min and then was blotted up with a piece of filter paper. At last, the sample was put at room temperature.

## Results and discussion

### Synthesis and characterization of the branched copolymers

**Fig. 1:**
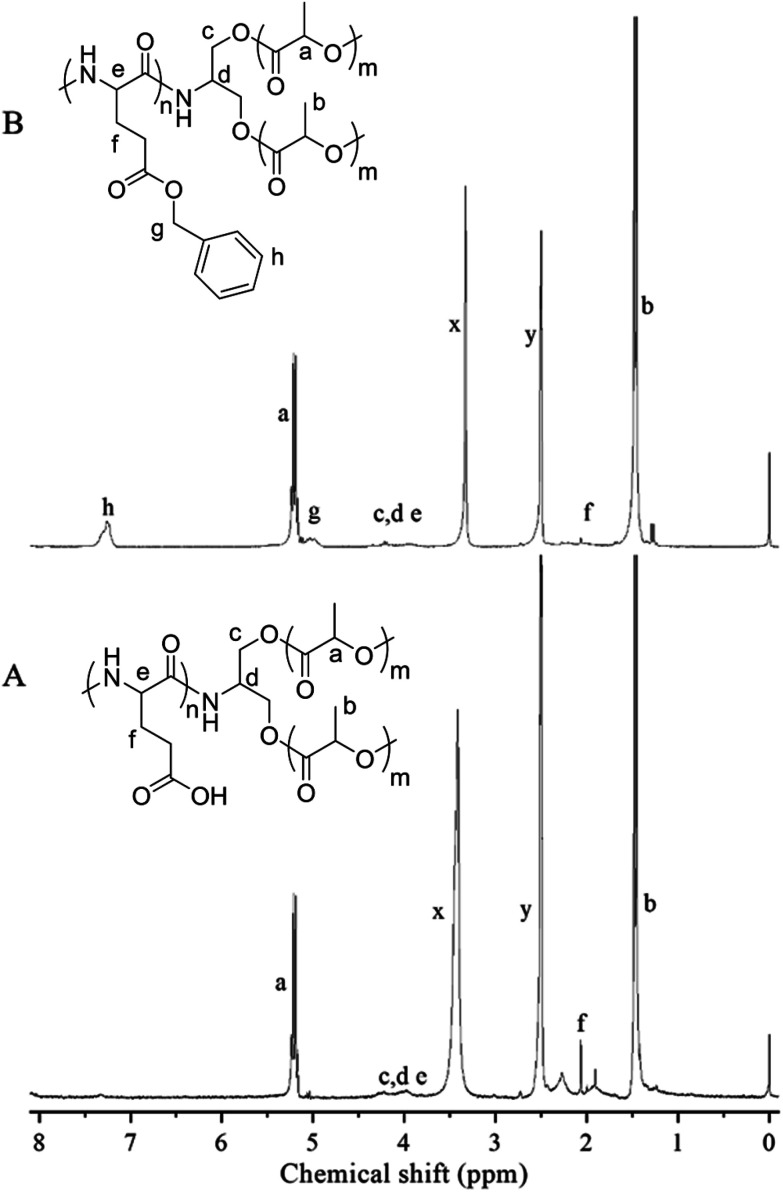
^1^H NMR spectra and their assignments of PLLA_2_–PBLG (B) and PLLA_2_–PLGA (A) in DMSO-*d*_6_ (*x* and *y* are solvent peaks).

**Fig. 2:**
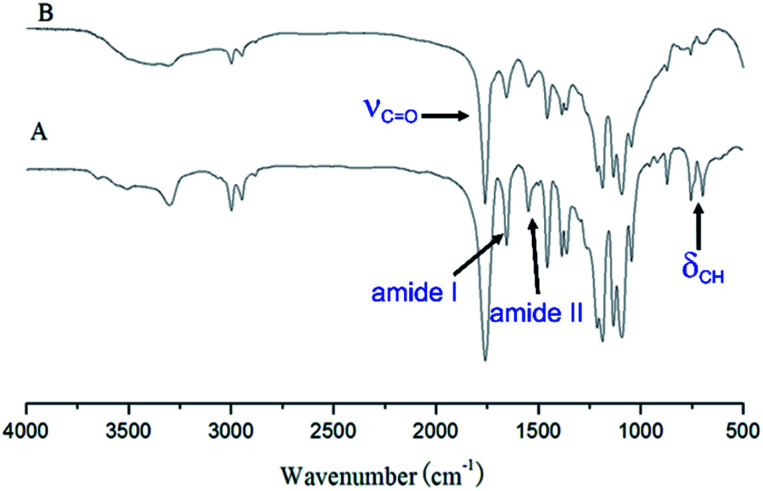
IR spectra of (A) PLLA_2_-*b*-PBLG, (B) PLLA_2_-*b*-PLGA.

**Fig. 3:**
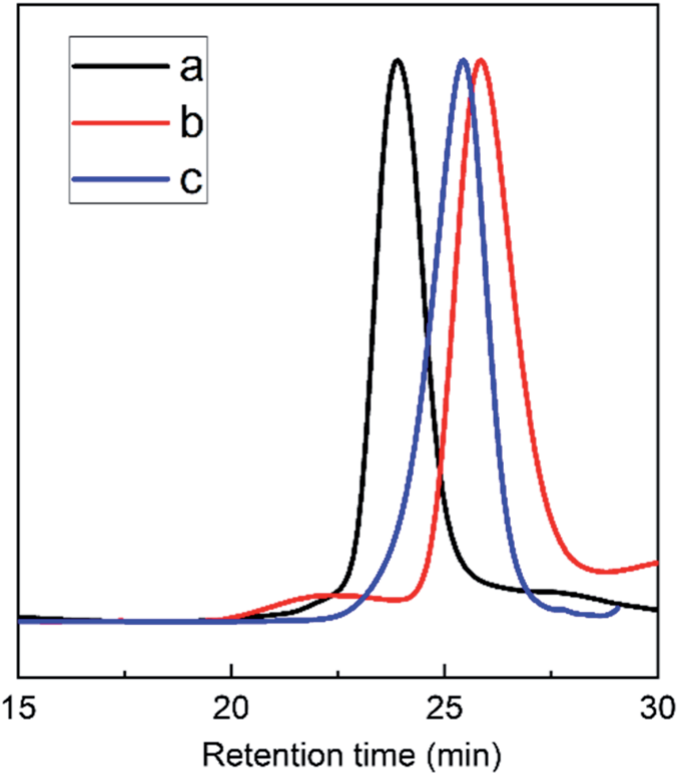
The GPC chromatographs of PLLA_2_–NH–Z (b), PLLA_2(30)_–PBLG_10_ (c) and PLLA_2(30)_–PBLG_55_ (a).

**Scheme 1:**
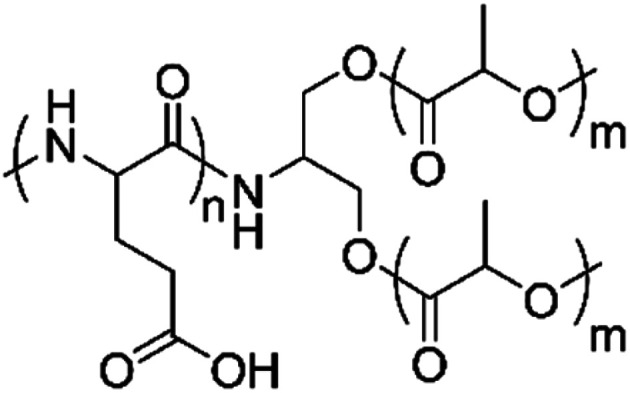
Structure of branched copolymer PLLA_2_–PGA.

The triblock copolymers were synthesized by a modified synthetic approach reported previously (Scheme S1†). The NH_2_–PLLA_2_ was obtained through ROP of l-lactide in the presence of stannous octoate with 2-amino-1,3-propanediol as an initiator in which the amine group was protected, and by subsequent deprotection. The third block was synthesized by ROP of *N*-carboxyanhydride of γ-benzyl l-glutamate with amino-terminated polylactide PLLA_2_–NH_2_ as a macroinitiator. All the peaks are well assigned. The degree of polymerization of PLLA (DPPLLA_2_ = 30) was obtained from the integral ratio of C_6_H_5_– in the initiator to –C(O)CH(CH_3_)O– in PLLA in the ^1^H NMR spectrum of PLLA_2_–NH–Z. Similarly, the DP_PBLG_ was calculated by the proton ratio of C_6_H_5_CH_2_OCO– and –C(O)CH(CH_3_)O–. The ^1^H NMR and the FTIR spectra are shown in Fig. 1B and 2A, respectively. The GPC traces of all samples show unimodal distribution (Fig. 3). This indicates well controlled polymerization of both PLLA and PLLA_2_–PBLG. Two of PLLA_2_–PBLG samples are synthesized, designated as PLLA_2(30)_–PBLG_55_ and PLLA_2(30)_–PBLG_10_ (numbers in the parentheses of the subscript designate degree of polymerization (DP), Table S1†) (Scheme 1).

The benzyl protective groups in poly(γ-benzyl-l-glutamic acid) can be removed by acidolysis with a 33% solution of HBr in HAc.^4^ The deprotection of PLLA_2_–PBLG is confirmed by as shown in Fig. 1A. The strong benzyl peak at 4.9 ppm and 7.2 ppm disappear in ^1^H NMR spectra. In the FTIR spectra, the *δ*_CH_ vibration of the benzyl at 749 and 697 cm^−1^ disappeared (Fig. 2). Both results suggest that the benzyl groups have been removed completely.^36^

### Self-assemble into the vesicles

The branched copolymer consists of both hydrophilic segment PLGA and hydrophobic segment poly(l-lactide) blocks. It thus can self-assembly into micelles in aqueous media. Moreover, due to the uncommon branched structure, the copolymer is expected to exhibit unique assembling property.

**Fig. 4:**
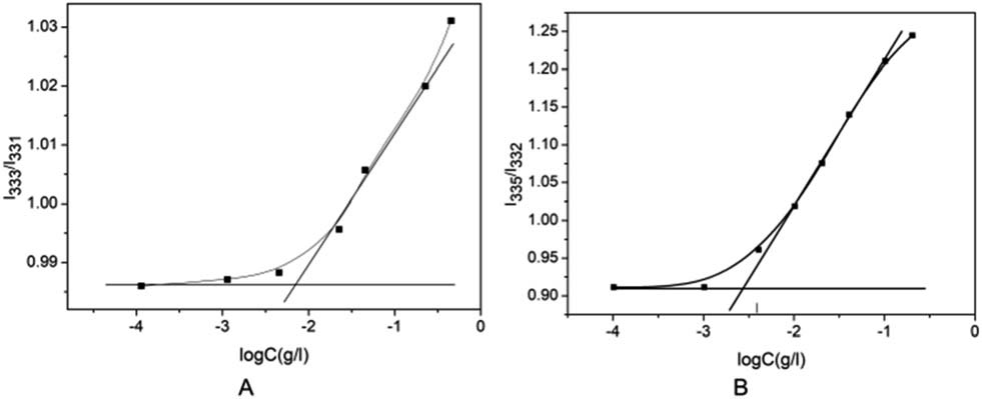
(A) Plot of *I*_333_/*I*_331_ of pyrene *vs.* log *C* of PLLA_2(30)_–PLGA_55_ in deionized water. (B) Plot of *I*_335_/*I*_332_ of pyrene *vs.* log *C* of PLLA_2(30)_–PLGA_10_ in deionized water.

**Fig. 5:**
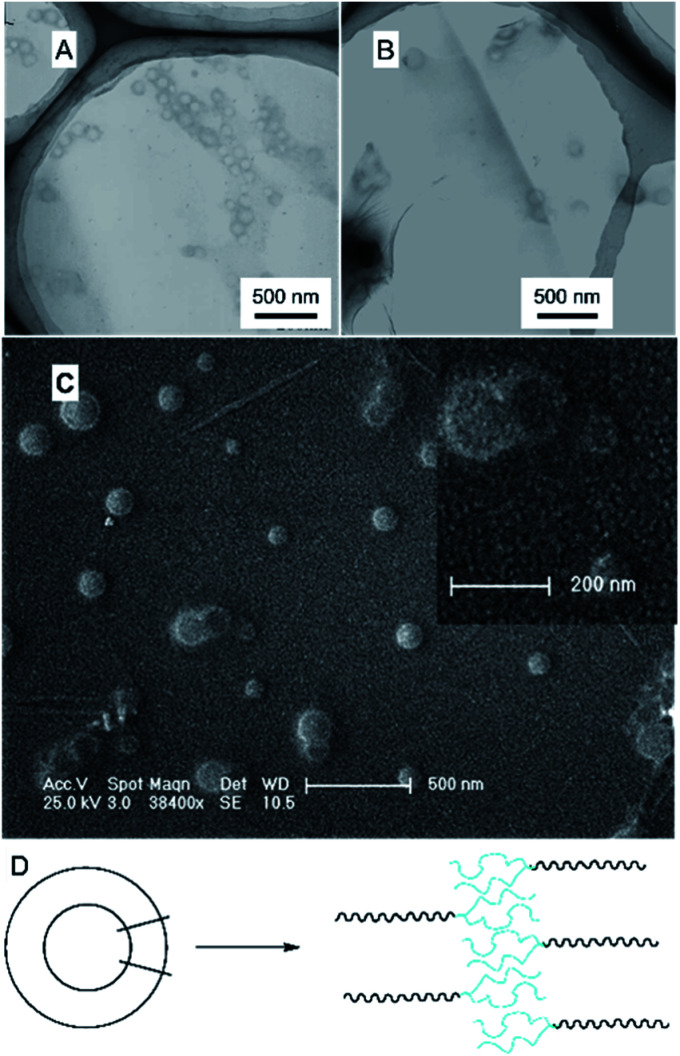
(A) TEM micrographs of the vesicles from PLLA_2(30)_–PLGA_55_. (B) TEM micrographs of the vesicles from PLLA_2(30)_–PLGA_10_. (C) ESEM micrographs of vesicles from PLLA_2(30)_–PLGA_55_, the inset is magnification. (D) Schematic representation of the vesicle.

**Fig. 6:**
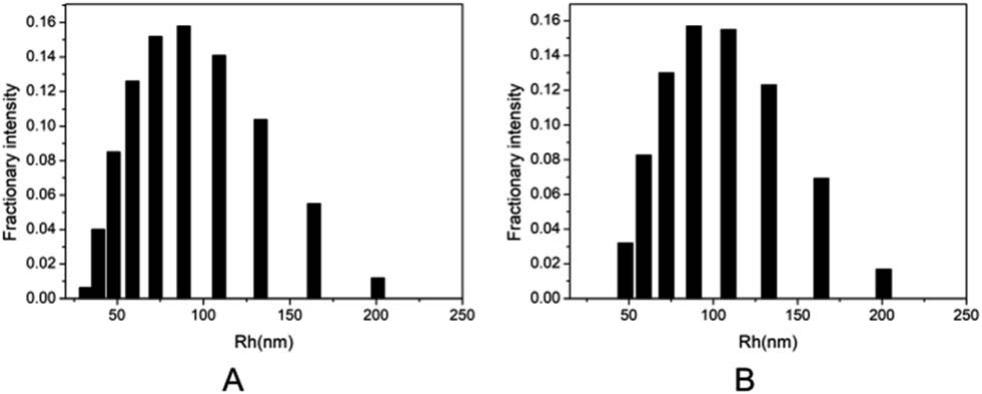
DLS graphs of the micelle size distribution of (A) PLLA_2(30)_–PLGA_55_ and (B) PLLA_2(30)_–PLGA_10_.

Two kinds of PLLA_2_–PLGA samples (PLLA_2(30)_–PLGA_55_ and PLLA_2(30)_–PLGA_10_) are employed for micelle preparation. Both samples have different chain lengths of hydrophobic block, but the same hydrophilic lengths. The micelle solutions were prepared by a process of solvent replacement. The formation of micelles is confirmed by a fluorescence technique using pyrene as a probe. The cmc value is obtained when the intensity ratio is plotted against the solution concentration (Fig. 4). The cmc values for the two samples PLLA_2(30)_–PLGA_55_ and PLLA_2(30)_–PLGA_10_ are 7.21 × 10^−3^ g l^−1^ and 2.62 × 10^−3^ g l^−1^ respectively. It can be seen that the cmc value decreases with the chain lengths of the hydrophobic segment increasing, consistent with the increasing hydrophobicity. The size and morphology of the micelles were further studied by TEM and DLS (Fig. 5 and 6). The TEM images show that both samples have uniform size with a spherical shape. The mean diameters are about 110.1 nm for PLLA_2(30)_–PLGA_55_ and 150.2 nm PLLA_2(30)_–PLGA_10_, respectively. The increased size with decreasing chain length of PLGA is possibly due to the reduced repulsive interaction of PLGA block. It can be seen that the darkness of the circumference is different from inside, which is compatible with characteristic of a hollow vesicle structure. All the particles are dispersed very well, and almost no cohesion happens during drying. This can be confirmed by DLS results. The hydrodynamic radius (RH) is calculated from the DLS data by the Stokes–Einstein equation, assuming that the micelles are of sphere shape.^37^ The average hydrodynamic radii (Rh) measured by DLS for PLLA_2(30)_–PBLG_55_ and PLLA_2(30)_–PBLG_10_ are 89.6 and 101.0 nm, respectively. However, the particle diameters measured by ESEM are a little smaller than hydrodynamic diameters measured by DLS. This may be because of the volume shrinkage during sample drying. The size distributions of vesicles are broad for the samples by DLS measurement (Fig. 6). However, the size of most vesicles is still close to the mean radii, consistent with that in TEM micrographs.

**Fig. 7:**
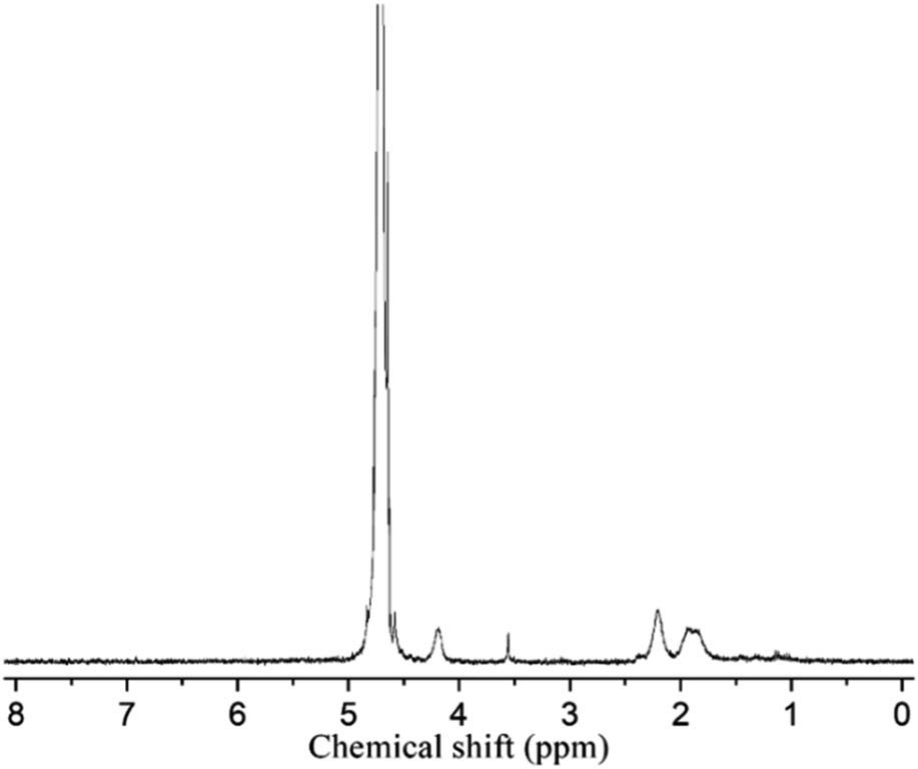
^1^H NMR spectra and their assignments of PLLA_2(30)_–PLGA_10_ in D_2_O.

To reveal the detailed structure of the vesicles, ^1^H NMR were performed in different solvents. As shown in Fig. 1A, all chemical shifts of three blocks are observed in the ^1^H NMR spectrum measured in DMSO-*d*_6_ which is a good solvent for the three blocks. The signals of the PLLA block at 1.4 and 5.2 ppm almost disappear in D_2_O (Fig. 7). The PLGA protons at 1.7–2.3 and 4.2 ppm are still observed. Note that the resonance peaks measured in D_2_O show different positions compared to those measured in DMSO-*d*_6_. This indicates that the PLLA block has left the water phase and PLGA segments remain solvated in water. Thus the insoluble PLLA block constitutes the middle-layer of the vesicle wall, while hydrophilic PLGA blocks form two outer-layers of the vesicle wall (Fig. 5D).

### Properties of the vesicles in solution

**Fig. 8:**
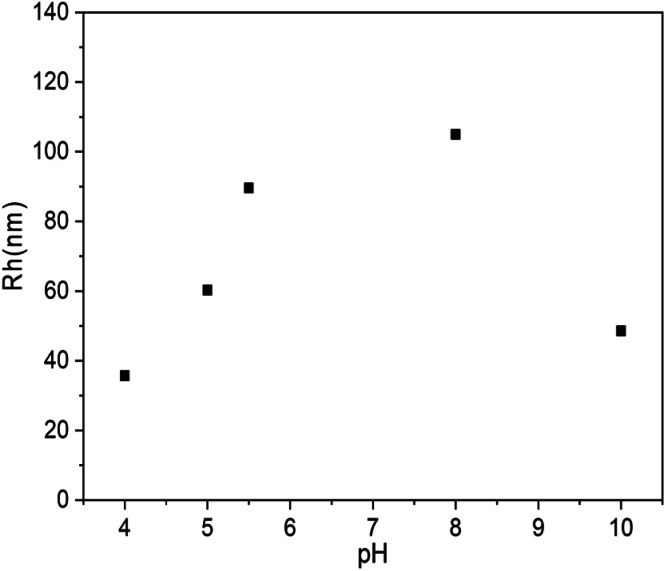
Plot of Rh of PLLA_2(30)_–PLGA_55_ vesicles *vs.* solution pH value (the concentration of the copolymer is 0.4 mg ml^−1^).

The above experiments confirmed the formation of vesicles from PLLA_2_–PLGA and its structure with PLGA as hydrophilic segments. It is known that there is a pendant carboxyl group on each LGA unit. These carboxyl groups can exist in acidic form or in salt form under proper conditions. The poly(l-glutamic acid) changes its charging status and molecular conformation at different pH. It assumes a α-helical conformation at a low pH and a coil conformation at a high pH.^38^ Thus, the self-assembled vesicle is expected to show pH-responsive property. 0.1 mol l^−1^ aqueous HCl or NaOH solution were added to the vesicles to obtain the solution with different pH values. The diameter of PLLA_2_–PLGA vesicles considerably increases with pH increasing as shown in Fig. 8. The copolymer PLLA_2(30)_–PBLG_55_ forms vesicles of 35.7 nm in Rh at pH 4 and of 105.6 nm at pH 8 at a concentration of 0.4 mg ml^−1^ in water. The PLLA block is non-ionized, it can not be sensitive to pH variation.

Thus, the dependence of the vesicle size is contributed by the pH-responsive property of PLGA block. As the COOH groups on the glutamic acid residues are deprotonated at high pH, the electrostatic repulsion forces between the deprotonated COOH groups are responsible for the dissolution of the PLGA segments and the stability of the entire vesicles in water. Moreover, the PLGA block adopts an extended coil conformation to decrease unfavorable side chain electrostatic repulsive forces at high pH. Thus, with the pH increasing, the PLGA block tends to be more stretched by the increasing electrostatic repulsive forces. The secondary conformation of PLGA converses from α-helix to random coil gradually. Furthermore, the strong repulsions of PLGA may lead to more flexible and less ordered hydrophobic walls constituted with PLLA. All these effects may result in the larger size vesicles to some extent. Similarly, as the solution pH decreases, the repulsive forces are weakened, which results in a process of desolvation, aggregation and an unstable system in water. In our system, when the pH of the solution is below 3, precipitation occurs. However, with the pH increasing to 10, the Rh of the vesicles shows decreasing trend. The block copolymer may form loose aggregation by strong electrostatic repulsion under basic condition. Thus, the aggregation number should be different from that of the vesicle prepared when solution pH < 10. This may be the reason for the shrinkage observed for the PLLA_2_–PLGA polymeric vesicles.^34^

**Fig. 9:**
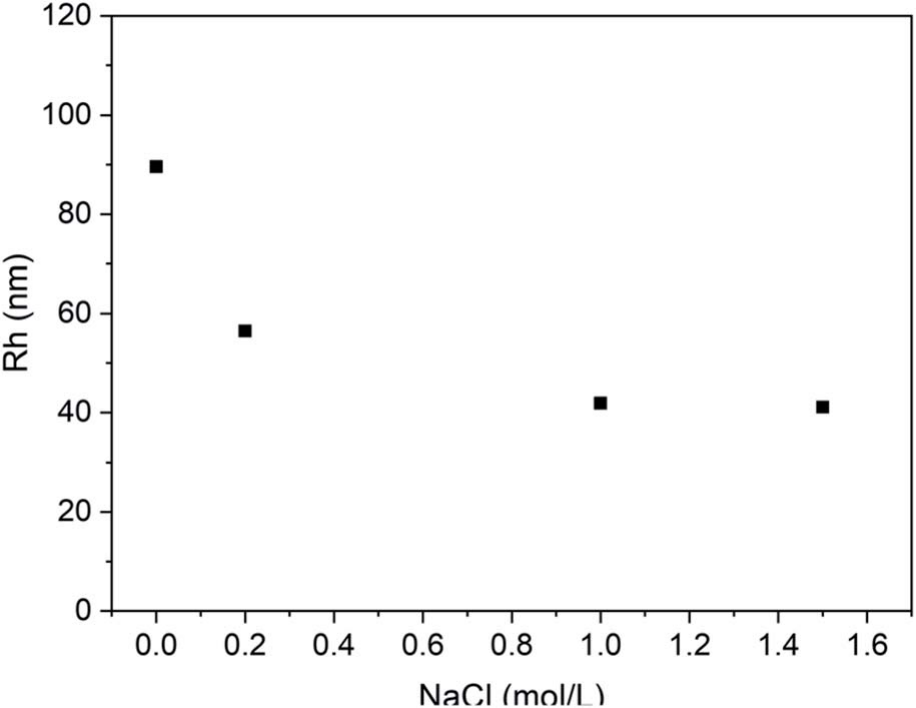
Plot of Rh of PLLA_2(30)_–PLGA_55_ vesicle *vs.* NaCl concentration at pH 5.5 (the concentration of the copolymer is 0.4 mg ml^−1^).

Furthermore, the effect of the ionic media on the vesicle solution was also investigated. As shown in Fig. 9, when the NaCl is added to the vesicle solution, the average vesicle size shows decreasing trend. This is mainly due to the shielding effect of the electrolytic ions on the charges of the PLGA residues. It can screen the repulsion between PLGA segments, leading to contraction of the vesicles in solution state.

**Fig. 10:**
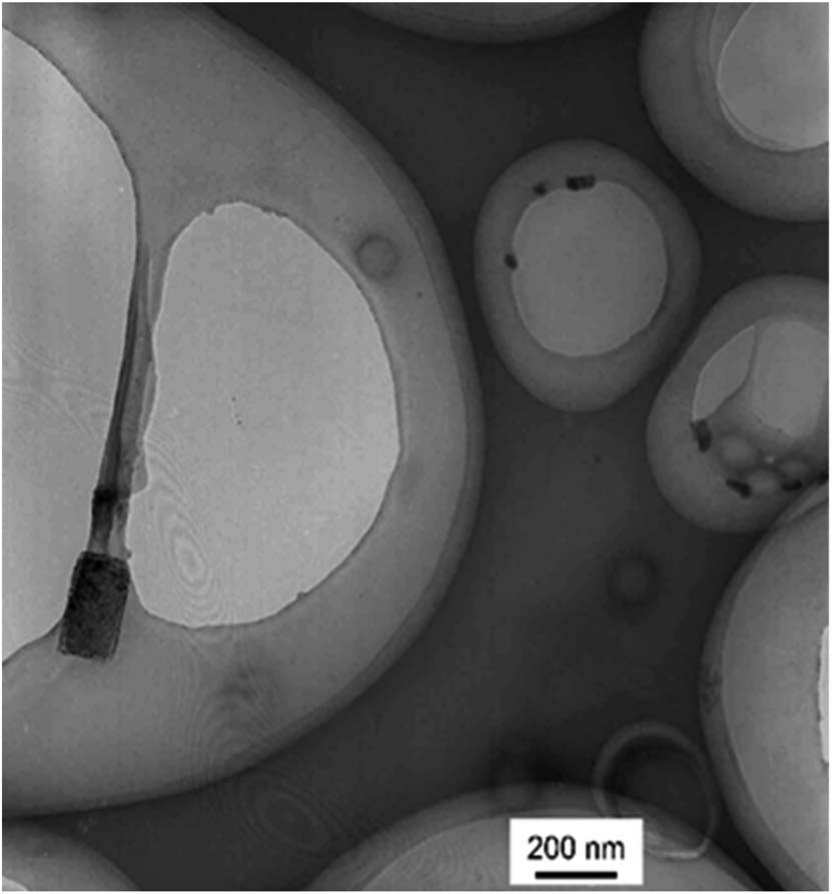
TEM micrographs of the lyophilized vesicles from PLLA_2(30)_–PLGA_55_.

For the purposes of testing the ability to withstand drying, redispersion of the polymeric vesicles after lyophilization is investigated. Fig. 10 shows the result of TEM measurements of redispersed polymeric vesicles in water. The average diameter of the redispersed polymeric vesicles was the same as that after dialysis. This result indicates that PLLA_2_–PLGA polymeric vesicles can be stored in a dry state.

## Conclusions

A new type of branched copolymer poly(l-lactide)_2_-*b*-poly(l-glutamic acid) (PLLA_2_–PLGA) has been synthesized, by acidolysis of the branched copolymer poly(l-lactide)_2_-*b*-poly(γ-benzyl-l-glutamic acid) (PLLA_2_–PBLG). The chemical structure of the block copolymer is confirmed by NMR, FT-IR, and GPC. Further, the self-assembly of the copolymers in aqueous media is described. It is observed that the vesicle is formed with insoluble PLLA block as the middle-layer of the vesicle wall, while hydrophilic PLGA blocks as the two outer-layers of the vesicle wall. The critical micelle concentration (cmc) of the copolymer is dependent on the ratio of the hydrophilic/hydrophobic segment. The detailed structure of the vesicles has been characterized with TEM, ESEM and NMR. The effects of pH value and salt concentration on the vesicle system were further studied. The vesicles during drying process remains after redispersing in aqueous media.

## Conflicts of interest

There are no conflicts to declare.

## Supplementary Material

RA-009-C9RA08703F-s001
