# Supplementary material for: pH-responsive polymeric vesicles from branched copolymers
Source: RSC Adv. 2019 Dec 11;9(70):41031–7. doi: 10.1039/c9ra08703f (PMC9076388; doi:10.1039/c9ra08703f)
Supplement: RA-009-C9RA08703F-s001 [file RA-009-C9RA08703F-s001.pdf]

Electronic Supplementary Material (ESI) for RSC Advances.
This journal is © The Royal Society of Chemistry 2019

Supporting information

pH-responsive polymeric vesicles from branched copolymers

Jinglun Zhou [1,3], Linlin Li, [2,*] Weishan Wang, [2] Yang Zhao, [2] and Shengyu Feng [1,*]

[1] Key Laboratory of Special Functional Aggregated Materials (Shandong University), Ministry of Education, School of Chemistry and Chemical Engineering, Shandong University, Jinan, 250100, China; tianzhuangzjl@126.com

[2] Shandong Institute for Product Quality Inspection, Jinan, 250100, China; wwshh1982@163.com (W.W.); 116102238@163.com (Y.Z.)

[3] Eco−Benign Plastics Technology Company Limited, Jinan, 250101, China; tianzhuangzjl@126.com

* Correspondence: fsy@sdu.edu.cn; lilinlin1003@163.com.

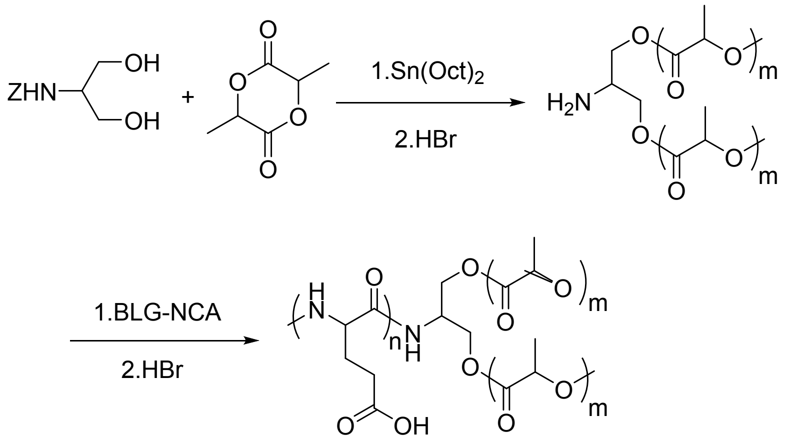

Scheme S1. Synthesis of branched copolymer $PLLA_2$-PBLG.

Table S1. Molecular weight characterization of the polymers

Polymers	feed ratio[a]	Mn[b] (kDa)	Mn[c] (kDa)	Disperity ($Đ$)[c]
$PLLA_2$-NH-Z	1:60	4.3	7.3	1.12
$PLLA_2$-NH_2	--	4.3	7.3	1.12
$PLLA_{2(30)}$-$PBLG_{10}$	60:15	6.5	9.7	1.21
$PLLA_{2(30)}$-$PBLG_{55}$	60:60	16.4	22.6	1.19

[a]Feed molar ratio of initiator/monomer. [b]Calculated from ^{1}H NMR spectra. [c]Determined from GPC.
